# A novel oncolytic adenovirus based on simian adenovirus serotype 24

**DOI:** 10.18632/oncotarget.15845

**Published:** 2017-03-02

**Authors:** Tao Cheng, Yufeng Song, Yan Zhang, Chao Zhang, Jieyun Yin, Yudan Chi, Dongming Zhou

**Affiliations:** ^1^ Vaccine Research Center, Key Laboratory of Molecular Virology and Immunology, Institut Pasteur of Shanghai, Chinese Academy of Science, Shanghai 200031, China

**Keywords:** oncolytic adenoviruses, AdC7, chimpanzee adenoviruses, p53-independent mitochondrial apoptosis, tumor treatment

## Abstract

Among the oncolytic virotherapy, an emerging treatment for tumor, adenoviruses are widely used at present in preclinical and clinical trials. Traditionally, oncolytic adenoviruses were developed based on the human adenovirus serotype 5 (AdHu5). However, AdHu5 has the drawbacks of preexisting anti-AdHu5 immunity in most populations, and extensive sequestration of Adhu5 by the liver through hexon, blood coagulation factor X (FX), and FX receptor interactions. To tackle these problems, we explored a novel oncolytic adenovirus AdC7-SP/E1A-ΔE3 for cancer treatment. AdC7-SP/E1A-ΔE3 was constructed by replacing the E1A promoter with tumor specific promoter survivin promoter and deleting E3 region using direct cloning methods based on simian adenovirus serotype 24 (namely AdC7). We showed that AdC7-SP/E1A-ΔE3 significantly killed tumor cell lines NCI-H508 and Huh7, and inhibited tumor growth in both NCI-H508 and Huh7 xenograft tumor models. Importantly, AdC7-SP/E1A-ΔE3 exhibited the antitumor efficacy via systemic administration. Mechanistically, infected cells were killed by AdC7-SP/E1A-ΔE3 via the p53-independent mitochondrial apoptosis pathway in which phosphorylation of BAD markedly declined and the expresses of Bik significantly went up. Therefore, AdC7-SP/E1A-ΔE3 is a promising candidate for liver and colon tumor treatment.

## INTRODUCTION

Cancer remains a leading cause of death worldwide. The complexity of tumor and the acquired or inherent resistance to treatments [1] contribute to less efficiency of traditional treatment of cancer, including surgery, chemotherapy and radiotherapy, which are not feasible for patients with advanced stage [2]. As a result, new treatments based on their unique mechanism of action are urgently needed. Cancer virotherapy, which employs a conditionally replicative virus, has attracted considerable attention, because it can kill cancer cells, but not normal cells, by selectively replicating in and transmitting among cancer cells [3]. Among oncolytic viruses currently explored to treat cancer, adenoviruses are the most widely used.

Oncolytic adenoviruses are engineered to selectively replicate in and kill tumor cells using different strategies. By virtue of its deleted E1B 55KDa gene, Onxy-015 was the first reported oncolytic adenovirus designed to replicate exclusively in p53-deleted or mutated cells [4]. The binding of EA1 to pRb protein through the CR2 region of E1A gene was required for adenovirus replication in normal cells rather than in tumor cells, and thus Ad5-Δ24 selectively replicate in tumor cells by deleting the CR2 region [5]. Oncolytic adenoviruses were also constructed by replacing viral promoters with tumor-specific promoters such as survivin promoter [6], PSA promoter [7], or telomerase promoter [8].

The most frequently used oncolytic adenoviruses are based on human adenovirus serotype 5, for its biology is well elucidated. However, preclinical and clinical trials have revealed several drawbacks of this serotype, one of which is widespread preexisting anti-AdHu5 immunity in humans due to prior infection in youth [9]. In addition, studies indicated that the hexon of AdHu5 was associated with blood coagulation factor X (FX), resulting in the direction of adenoviruses to FX receptors abundantly expressed in liver cells, which caused massive transduction to the liver [10, 11]. Recently, some low-seroprevalence human adenoviruses or adenoviruses originated from other species such as chimpanzee were modified into viral vectors, to circumvent preexisting anti-Adhu5 immunity [12, 13]. Importantly, some of these adenoviruses, such as AdHu26 and AdHu48, did not bind to FX [14], leading to diminished transduction to the liver. AdHu5 and chimpanzee adenoviruses are antigenically distinct; AdHu5-neutralizing antibodies could hardly influence the chimpanzee adenoviruses-derived vector, which enabled simian-derived adenoviral vectors to become promising vector prototypes [15].

AdC7, originated from chimpanzee, circulates rarely in human populations, and it is not subject to neutralization by preexisting anti-AdHu5 antibodies, as confirmed by previous studies [15, 16]. Compared to AdHu5-derived oncolytic adenoviruses, AdC7-originated oncolytic adenoviruses have a great potential to diminish liver transduction, because the FX-AdC7 complexes are unstable [17]. An oncolytic AdC7 platform therefore has some advantages over an AdHu5 platform. AdC7 was initially constructed into a replication-deficient vector, which was used to develop a great variety of vaccines including (but not limited to) HIV vaccines [18], Ebola vaccines [19] and influenza vaccines [16, 20]. To our knowledge, that chimpanzee adenoviruses are engineered into oncolytic viruses has not been previously reported. In this study, we constructed the oncolytic chimpanzee adenovirus AdC7-SP/E1A-ΔE3, in which the E1 promoter was replaced by the tumor-specific survivin promoter and the E3 region was deleted. These recombinant adenoviruses could replicate in many types of cancer cell lines, including NCI-H508, Huh7, A549, and SiHa. Moreover, AdC7-SP/E1A-ΔE3 could efficaciously kill NCI-H508 and Huh7 *in vitro* and *in vivo*, indicating that AdC7-SP/E1A-ΔE3 has the potential to treat such cancers.

## RESULTS

### E1A expression driven by survivin promoter in tumor cell lines

**Figure 1:**
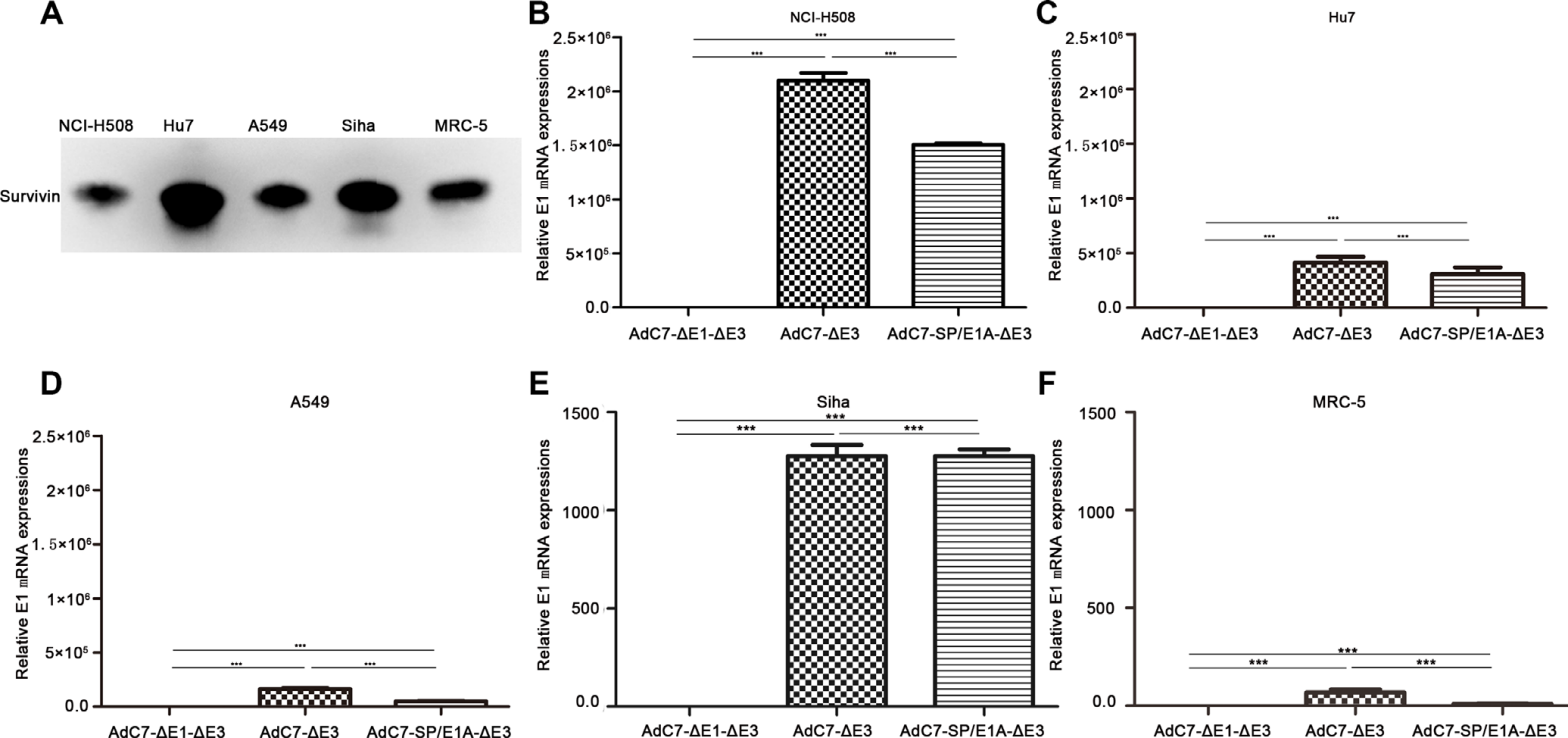
Expression of survivin and E1A genes in different cell types (**A**) Western blotting was performed to detect survivin proteins in 1 × 10^6^ cells. (**B**–**F**) A panel of NCI-H508 (B), Huh7 (C), A549 (D), SiHa (E), and MRC-5 (F) cells was infected with AdC7-ΔE1-ΔE3, AdC7-ΔE3, and AdC7-SP/E1A-ΔE3 at 10 MOI. E1A mRNA was quantitated by real-time PCR at 24 h post infection. Relative levels of E1A mRNA were shown in reference to GAPDH expression; relative E1 mRNAs in cells infected with AdC7-ΔE1-ΔE3 were defined as 1. Data shown are means ± standard errors of the means (SEM); statistical significance was determined by one-way ANOVA: ***P < 0.0001. Each experiment was performed three times.

Survivin, a small member of the inhibitor of apoptosis family, is expressed in tumor cells and fetal tissues but not in terminally differentiated cells, and thus is considered a potential target in tumor treatment [21]. Previous studies indicated that survivin promoter is a tumor-specific promoter, and that a wide range of tumor cells express the survivin protein [6]. The present study used western blotting to assay the expression of survivin in four tumor cell lines and a normal cell line. As shown in Figure 1A, all four tumor cell lines (∼1 × 10^6^ cells) expressed the survivin protein, indicating that they each have survivin promoter activity. In addition, normal MRC-5 cells expressed the survivin protein.

AdC7-SP/E1A-ΔE3 is an oncolytic adenovirus in which E1A expression is driven by a survivin promoter and the E3 region is deleted; AdC7-ΔE3 is a modified adenovirus without the E3 region; and AdC7-ΔE1-ΔE3 is a non-replicating adenovirus lacking the E1 and E3 regions. Strong activity of survivin promoter in various types of tumor cell has been confirmed by previous studies [12, 22]. To explore the expression of E1A by AdC7-SP/E1A-ΔE3, we infected a panel of tumor cell lines with AdC7-SP/E1A-ΔE3. Figure 1B–1E show that tumor cell lines (NCI-H508, Huh7, A549, and SiHa) infected with AdC7-SP/E1A-ΔE3 exhibited significant increases in E1A expression relative to counterparts infected with AdC7-ΔE1-ΔE3 (*p* < 0.0001). As shown in Figure 1F, MRC-5 cells expressed considerably less E1A mRNA when infected with AdC7-SP/E1A-ΔE3 than when infected with AdC7-ΔE3 (*p* < 0.0001).

### Efficient replication of AdC7-SP/E1A-ΔE3 in tumor cells

**Figure 2:**
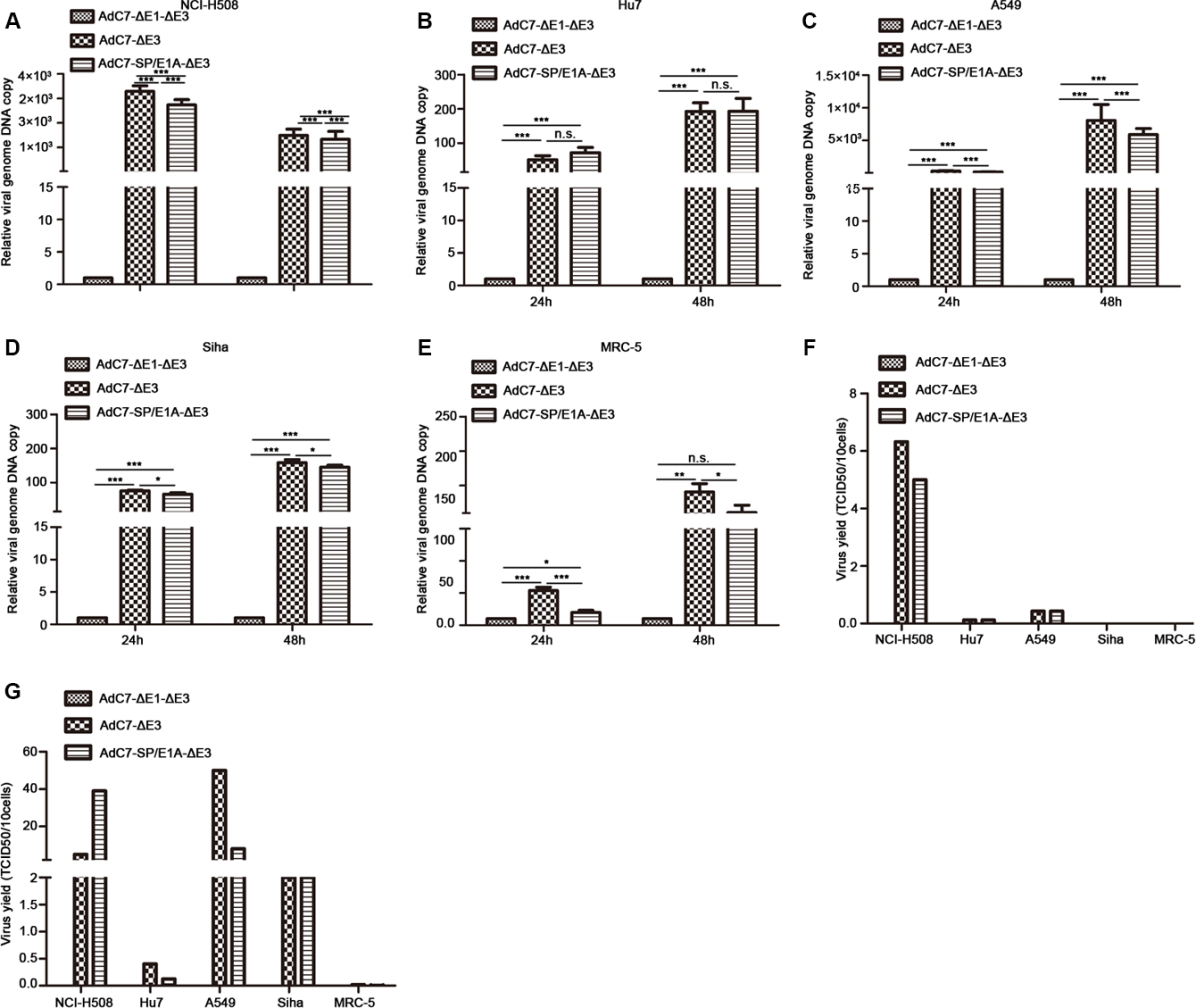
Replication of AdC7-ΔE1-ΔE3 in a panel of cells (**A**–**E**) A panel of NCI-H508 (A), Huh7 (B), A549 (C), SiHa (D), and MRC-5 (E) cells was infected with AdC7-ΔE1-ΔE3, AdC7-ΔE3, and AdC7-SP/E1A-ΔE3 at 10 MOI, and sampled at different times post-infection. Relative viral genome copies were determined through real-time PCR. GAPDH was used as the reference and viral copies was defined as 1 in cells infected with AdC7-ΔE1-ΔE3. Data shown are means ± standard errors of the means (SEM); statistical significance was determined by one way ANOVA: ***P < 0.0001. Each experiment was performed three times. (**F**, **G**) A panel of NCI-H508, Huh7, A549, SiHa, and MRC-5 cells was infected with AdC7-ΔE1-ΔE3, AdC7-ΔE3, and AdC7-SP/E1A-ΔE3 at 10 MOI, and was twice collected and washed with PBS, at 24 h (F) and 48 h (G) post-infection. After three freeze-thaw cycles, progeny virus was quantitated with the TCID_50_ assay. Data shown are mean of two independent experiments.

Having found that E1A was expressed in tumor cells infected with AdC7-SP/E1A-ΔE3, we speculated that AdC7-SP/E1A-ΔE3 was efficiently replicated in infected tumor cells, because E1A is required for adenovirus replication. To test this hypothesis, a panel of tumor cells was infected, at 10 MOI, with AdC7-ΔE1A-ΔE3, AdC7-ΔE3, or AdC7-SP/E1A-ΔE3, and relative viral genome copy numbers, which served as the readout for viral replication, were detected by RT-PCR. As shown in Figure 2A–2D, we confirmed that AdC7-SP/E1A-ΔE3 could replicate in a panel of tumor cell lines (NCI-H508, Huh7, A549, or SiHa); relative viral genome copy numbers were significantly higher in cells infected with AdC7-SP/E1A-ΔE3 than in cells infected with AdC7-ΔE1A-ΔE3 (*p* < 0.0001). Due to the deletion of the E1 region, AdC7-ΔE1A-ΔE3 is a non-replicating adenovirus. When infected with AdC7-SP/E1A-ΔE3, MCR-5 cells had dramatically lower viral copy numbers than when infected with AdC7-ΔE3, as shown in Figure 2E. To assay the replication competence of AdC7-SP/E1A-ΔE3 more accurately in tumor cells, progeny viruses produced in tumor cells were quantitated by TCID_50_ assay, after the infection of cells with adenoviruses at 10 MOI. As shown in Figure 2F, at 24 h after infection with AdC7-ΔE3 or AdC7-SP/E1A-ΔE3, adenoviruses were detectable in tumor cells but not in MRC-5 cells. In the NCI-H508 tumor cells, infected with AdC7-SP/E1A-ΔE3, 10 cells could yield 5 TCID_50_ of adenoviruses; the NCI-H508 cells produced 10-700 fold more progeny viruses than all other tumor cells. Figure 2G suggests that at 48 h post infection, tumor cell lines A549 and SiHa could produce more noticeable numbers of the progeny virus, and Huh7 cells produced the least virus among all tested tumor cell lines. While adenoviruses were detectable in MRC-5 cells infected with AdC7-SP/ E1A-ΔE3, only 0.006 TCID_50_ of progeny viruses were produced in ten of these infected MRC-5 cells. The dose of progeny viruses in MCR-5 was decreased three-fold when infected with AdC7-SP/ E1A-ΔE3, compared to when infected with AdC7-ΔE3.

### Tumor cytotoxicity of AdC7-SP/E1A-ΔE3 *in vitro*

**Figure 3:**
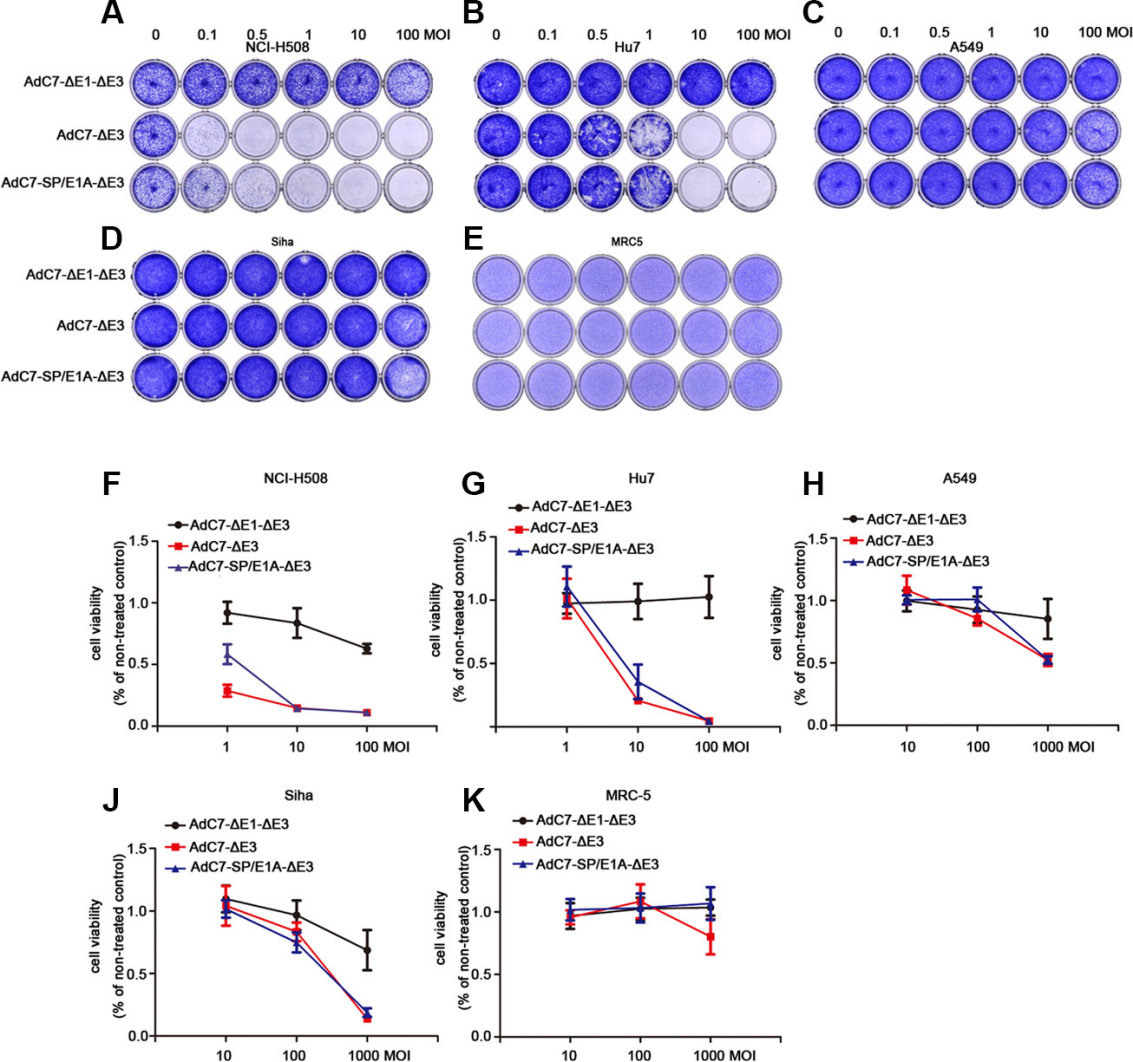
AdC7-ΔE1-ΔE3 induced cytotoxicity of tumor cells A panel of NCI-H508, Huh7, A549, SiHa, and MRC-5 cells was infected with AdC7-ΔE1-ΔE3, AdC7-ΔE3, and AdC7-SP/E1A-ΔE3 at various MOIs. At 5 d post infection (**A**–**E**), cells were stained with crystal violet. Graph represents one of three different experiments. (**F**–**K**) Infected cells were subject to MTT assay kat 5 d post infection. Data are presented as means ± SEM of triplicate samples, and are representative of three independent experiments.

To assay the cytopathic effects (CPE) of AdC7-SP/E1A-ΔE3 on tumor cells, crystal violet cell staining assay was performed. A panel of tumor cells was stained with crystal violet 5 d after infection with AdC7-ΔE1A-ΔE3, AdC7-ΔE3, or AdC7-SP/E1A-ΔE3, at MOIs from 0.1–100. As shown in Figure 3A–3D, AdC7-SP/E1A-ΔE3 induced a prominent cytotoxic effect on NCI-H508 cells even when infection occurred at an MOI of 0.1; moreover, this virus decisively killed Huh7 cells at an MOI of 1. Although AdC7-SP/E1A-ΔE3 could replicate in A549 cells and SiHa cells (Figure 2), its cytotoxic effects on them were modest even at an MOI of 100. Figure 3E shows that AdC7-SP/E1A-ΔE3 had no cytotoxic effects on MRC-5 cells at 5 d post-infection. To further quantify the cytotoxic effects induced by AdC7-SP/E1A-ΔE3, cell viability was examined through MTT assay, which was carried out 3 d post-infection with three different adenoviruses at various MOIs. As shown in Figure 3F–3K, NCI-H508 cells and Huh7 cells were sensitive to adenoviral cytotoxicity at 10 MOI, while A549 and SiHa cell lines did not exhibit obvious cytotoxicity even at extremely high MOI (1000). Among all tested tumor cells, NCI-H508 cells were the most sensitive to AdC7-SP/E1A-ΔE3; AdC7-SP/E1A-ΔE3 killed these cells even at an MOI of 1. In addition, Figure 3K shows that AdC7-SP/E1A-ΔE3 was less cytotoxic than AdC7-ΔE3 in MRC-5 cell lines.

### AdC7-SP/E1A-ΔE3 kills tumor cells through p53-independent mitochondrial apoptosis pathway

**Figure 4:**
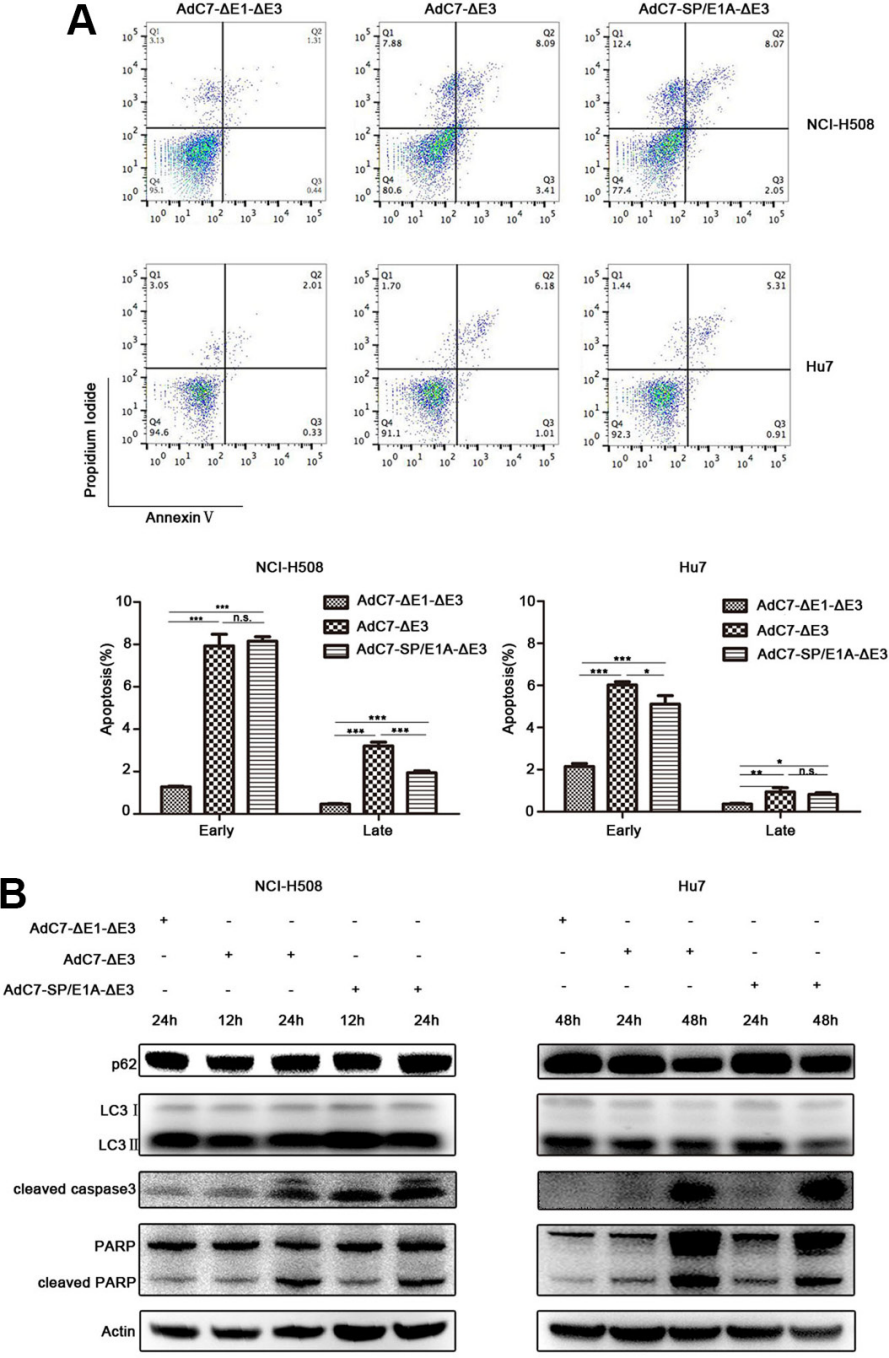
AdC7-ΔE1-ΔE3 trigger tumor cell apoptosis (**A**) NCI-H508 and Huh7 cells were infected at 100 MOI with AdC7-ΔE1-ΔE3, AdC7-ΔE3, and AdC7-SP/E1A-ΔE3. Two cells were collected 24 h and 48 h post infection respectively. Flow cytometry was performed to evaluate tumor cell apoptosis after staining NCI-H508 and Huh7 with propidium iodide (PI)/annexin V. Early apoptosis: AV^+^/PI^−^ (%); later stage apoptosis: AV^+^/PI^+^ (%). Data are presented as means ± SEM of triplicate samples, and are representative of three independent experiments. Statistical significance was determined by one way ANOVA: ***P < 0.0001, **< 0.001, *< 0.05. (**B**) NCI-H508 and Huh7 cells were infected at 100 MOI with AdC7-ΔE1-ΔE3, AdC7-ΔE3, and AdC7-SP/E1A-ΔE3. NCI-H508 cells were collected at 12 h and 24 h post infection, and Huh7 cells were collected 24 h and 48 h post infection. Western blotting was carried out to detect levels of p62, LC3, cleaved caspase3, and cleaved PARP in NCI-H508 and Huh7 cells. β-actin was used as a loading control.

To explore the mechanism by which AdC7-SP/E1A-ΔE3 killed NCI-H508 and Huh7 cells, we assayed apoptosis of NCI-H508 and Huh7 through flow cytometry after AdC7-SP/E1A-ΔE3 infection. As shown in Figure 4A, AdC7-SP/E1A-ΔE3 could trigger NCI-H508 and Huh7 apoptosis because they were labeled by annexin V alone (early apoptosis), or both annexin V and PI (late apoptosis). To confirm the apoptosis that occurred in NCI-H508 and Huh7 cells, we used western blotting to examine the apoptosis inducers, cleaved caspase 3 and PARP. The data shown in Figure 4B indicated that cleaved caspase 3 and PARP were augmented in NCI-H508 and Huh7 cells infected with AdC7-SP/E1A-ΔE3. Since Adhu5 killed cells via autophagic cell death [23], AdC7-SP/E1A-ΔE3 is also likely to have induced autophagic cell death in NCI-H508 and Huh7 cells. Contrary to this hypothesis, autophagic cell death was not detectable in NCI-H508 and Huh7 cells infected with AdC7-SP/E1A-ΔE3; levels of p62 did not decrease, and levels of LC3 II did not increase (Figure 4B).

**Figure 5:**
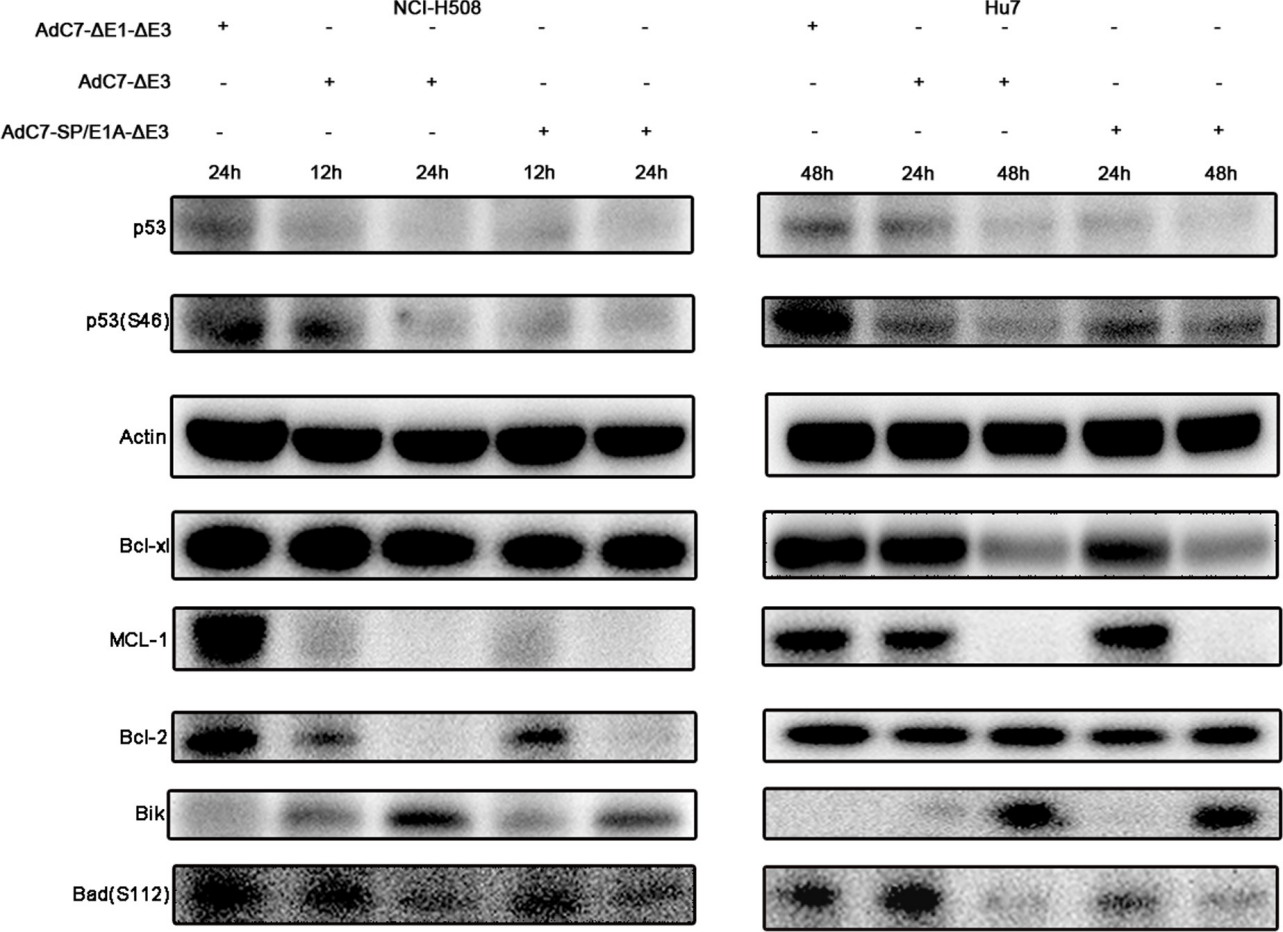
Tumor cell apoptosis is induced by AdC7-SP/E1A-ΔE3 via a p53-independent mitochondrial pathway NCI-H508 and Huh7 cells were infected at 100 MOI with AdC7-ΔE1-ΔE3, AdC7-ΔE3, and AdC7-SP/E1A-ΔE3. NCI-H508 cells were collected at 12 h and 24 h post infection, and Huh7 cells were collected 24 h and 48 h post-infection. Western blotting was carried out to detect levels of p53, phosphorylation of p53, MCL-1, Bcl-2, Bcl-xl, Bik and phosphorylation of Bad in NCI-H508 and Huh7 cells. β-actin was used as a loading control.

Previous studies [24], in which the E1B-55K and E4-25K proteins of AdHu5 were associated with other cellular proteins to form a ubiquitin ligase complex that polyubiquitinated and degraded p53, implied that AdC7-SP/E1A-ΔE3 also degraded p53. This hypothesis was validated by the data in Figure 5, where p53 levels were markedly lowered in cells infected with AdC7-SP/E1A-ΔE3. These diminished p53 levels indicate that AdC7-SP/E1A-ΔE3 induced apoptosis in a p53-independent manner. It was further validated by the fact that the phosphorylation of p53 at Ser46, which induces the expression of relevant apoptosis genes, was significantly decreased in cells infected with AdC7-SP/E1A-ΔE3 (Figure 5). NCI-H508 infected with AdC7-SP/E1A-ΔE3 exhibited marked drops in levels of pro-survival proteins MCL-1and Bcl-2, while Huh7 exhibited marked drops in levels of pro-survival proteins MCL-1and Bcl-xl (Figure 5). We also note that both NCI-H508 and Huh7 cells have noticeably diminished levels of Bad phosphorylation at serine 112 and have increased expression of Bik (Figure 5).

### AdC7-SP/E1A-ΔE3 inhibits tumor growth in xenograft tumor models

**Figure 6:**
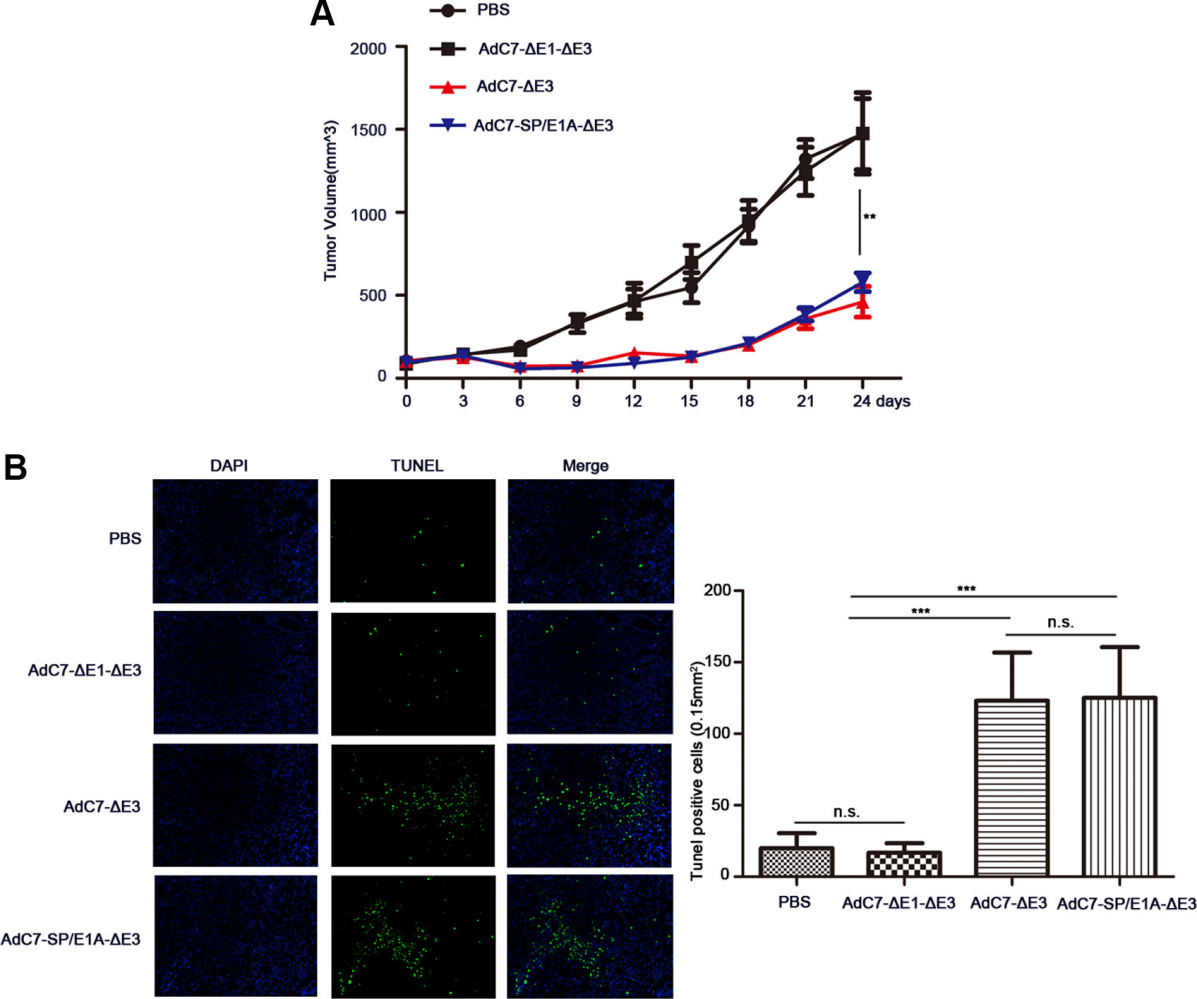
AdC7-SP/E1A-ΔE3 inhibit tumor growth in nude mouse NCI-H508 cell xenografts NCI-H508 cells (10^7^ ) were inoculated into the right flanks of nude mice, and tumor was injected with 5 × 10^8^ PFU adenovirus (AdC7-ΔE1-ΔE3, AdC7-ΔE3, or AdC7-SP/E1A-ΔE3) suspended in 50 μL of PBS or 50 μL PBS alone when reaching 100–150 mm^3^. (**A**) Tumor volume (*n* = 6) was measured every three days. (**B**) In left images, TUNEL staining of tumor cells, and in right images, TUNEL positive cells (*n* = 5) were quantitated. Data are presented as means ± SEM. Statistical significance was determined by one way ANOVA: ***< 0.0001, **< 0.001. Data are representative of two independent experiments.

**Figure 7:**
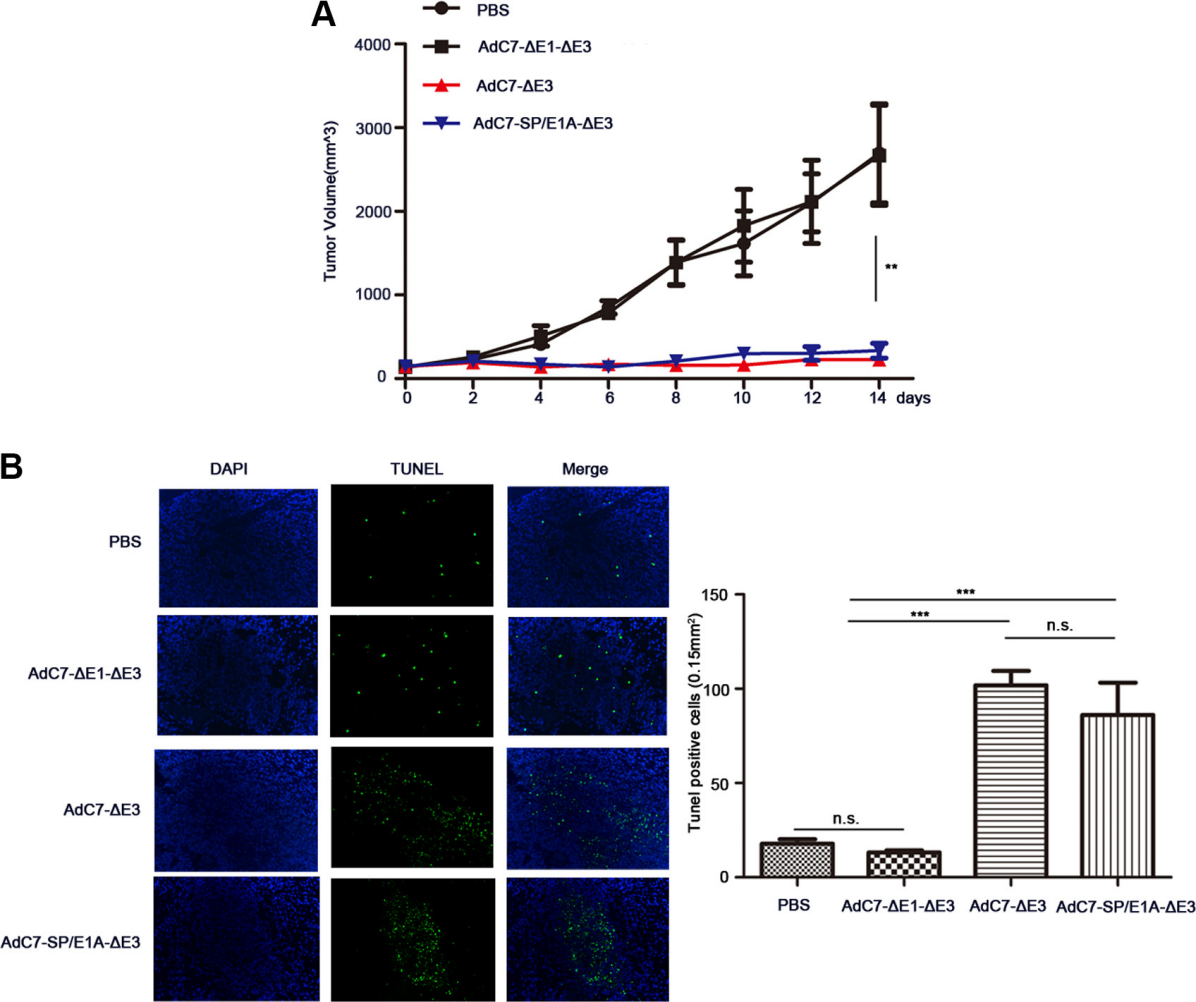
AdC7-SP/E1A-ΔE3 inhibit tumor growth in nude mouse Huh7 cell xenografts Huh7 cells (5 × 10^6^) were inoculated into the right flanks of nude mice, and tumor was injected with 5 × 10^8^ PFU adenovirus (AdC7-ΔE1-ΔE3, AdC7-ΔE3, or AdC7-SP/E1A-ΔE3) suspended in 50 μL of PBS or 50 μL PBS alone when reaching 100-150 mm^3^. (**A**) Tumor volume (*n* = 6) was measured every two days. (**B**) In left images, TUNEL staining of tumor cells, and in right images, TUNEL positive cells (*n* = 5) were quantitated. Data are presented as means ± SEM. Statistical significance was determined by one way ANOVA: ***< 0.0001, **< 0.001. Data are representative of two independent experiments.

To examine the antitumor activity of AdC7-SP/E1A-ΔE3 *in vivo*, xenograft mouse tumor models were established. We inoculated matrigel mixtures of NCI-H508 or Huh7 tumor cells under the skin; when the tumor volume reached 100-150 mm^3^, we treated them with PBS, AdC7-ΔE1A-ΔE3, AdC7-ΔE3, or AdC7-SP/E1A-ΔE3 through intratumoral injection. As shown in Figure 6A, in NCI-H508 xenograft models, AdC7-ΔE3 and AdC7-SP/E1A-ΔE3 groups induced dramatic declines in tumor volume relative to control groups (PBS group, AdC7-ΔE1A-ΔE3), while AdC7-ΔE3 and AdC7-SP/E1A-ΔE3 groups had no significant difference in tumor volume. In Figure 6B, the number of TUNEL-positive cells was considerably more in AdC7-ΔE3 and AdC7-SP/E1A-ΔE3 groups than in the control groups (*p* < 0.0001). Similarly, the Huh7 xenograft experiments showed that AdC7-ΔE3 and AdC7-SP/E1A-ΔE3 significantly inhibited tumor growth (Figure 7A) by triggering tumor cell apoptosis, which was validated by TUNEL staining assay (Figure 7B).

### Antitumor efficacy of AdC7-SP/E1A-ΔE3 via systemic administration

**Figure 8:**
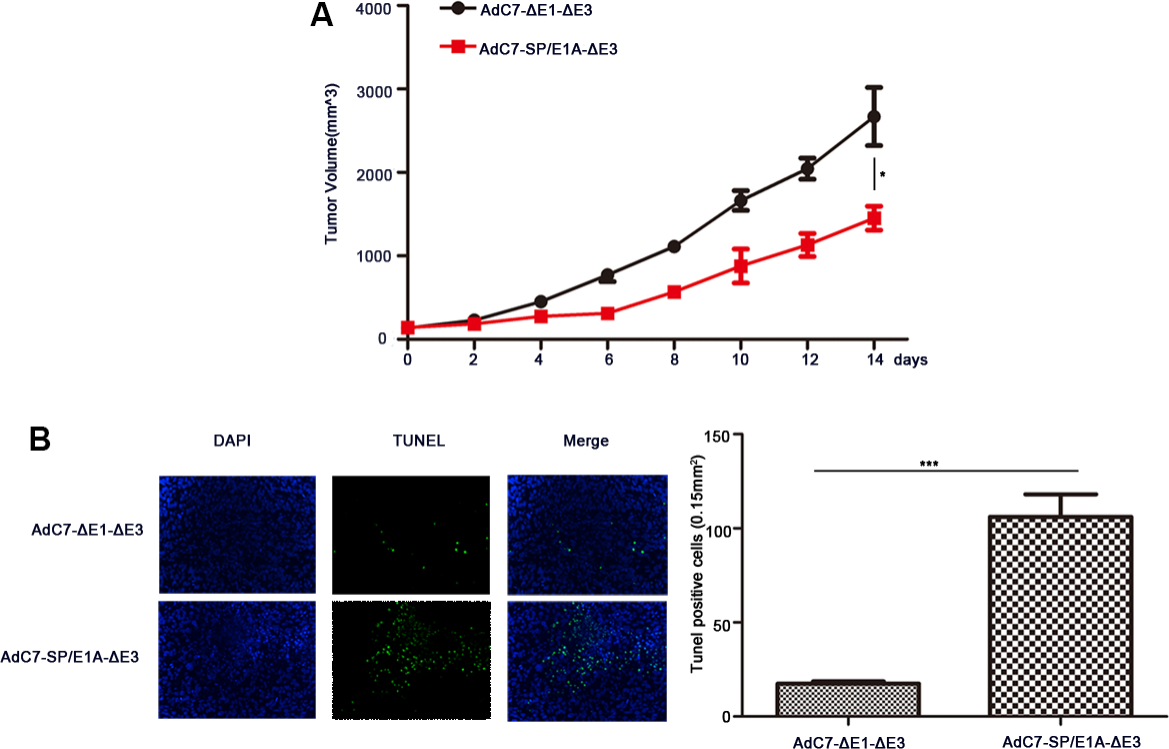
The antitumor efficacy of AdC7-SP/E1A-ΔE3 via systemic administration Huh7 cells (5 × 10^6^) were inoculated into the right flanks of nude mice, and tumor was injected with 1 × 10^9^ PFU adenovirus (AdC7-ΔE1-ΔE3 or AdC7-SP/E1A-ΔE3) suspended in 50 μL of PBS three times at the interval of one day when reaching 100–150 mm^3^. (**A**) Tumor volume (*n* = 6) was measured every two days. (**B**) In left images, TUNEL staining of tumor cells, and in right images, TUNEL positive cells (*n* = 5) were quantitated. Data are presented as means ± SEM. Statistical significance was determined by student's *t* test: ***< 0.0001, *< 0.05. Data are representative of two independent experiments.

To investigate the antitumor activity induced by AdC7-SP/E1A-ΔE3 via intravenous injection, we inoculate the mixture of Huh7 cells with matrigel under the skins in the xenograft mouse tumor models; when tumors reached 100–150 mm^3^, mice were intravenously injected with 1 × 10^9^ PFU of AdC7-ΔE1A-ΔE3 or AdC7-SP/E1A-ΔE3. As shown in Figure 8A, the tumor volume of mice treated with AdC7-SP/E1A-ΔE3 was 1.8 fold smaller than that of mice injected with AdC7-ΔE1A-ΔE3 (*p* < 0.05). Furthermore, immunohistofluorescence indicated that the number of TUNEL positive tumor cells in the injected group with AdC7-SP/E1A-ΔE3 was 7.0 fold more than in the group treated with AdC7-ΔE1A-ΔE3(*p* < 0.0001) (Figure 8B).

## DISCUSSION

To circumvent preexisting anti-AdHu5 immunity in populations, chimpanzee adenoviruses have been engineered into replication-deficient vaccine vectors [25]. These replication-deficient vectors have been used to evaluate a wide range of vaccines, such as HIV, Ebola, and rabies virus vaccines in preclinical and clinical trials [26–29]. Like other chimpanzee adenoviruses, AdC7 is not neutralized by anti-Adhu5 antibodies and rarely circulates in human populations [15, 16]. Unlike the hexon protein of AdHu5, AdC7 hexon is not associated with FX [17], indicating that AdC7 does not bind FX receptors expressed abundantly in liver cells, thus avoiding extensive liver sequestration of adenoviruses. Therefore, in this study, conditionally replicating AdC7-SP/E1A-ΔE3, in which the replication of AdC7-SP/E1A-ΔE3 in cells was dependent on survivin promoter activity, was constructed to tackle the drawbacks of AdHu5-based oncolytic adenoviruses: preexisting anti-AdHu5 immunity in most populations, and entry of AdHu5 into liver cells via FX factor. Although AdC7 has some advantages over AdHu5 as oncolytic viruses, AdC7 without modification could not address the AdHu5 drawback of nonselectivity in infecting target cells. This problem might be overcame by attempts to genetically alter the fiber protein [30] or to insert some peptides into the hexon region [31].

**Figure 9:**
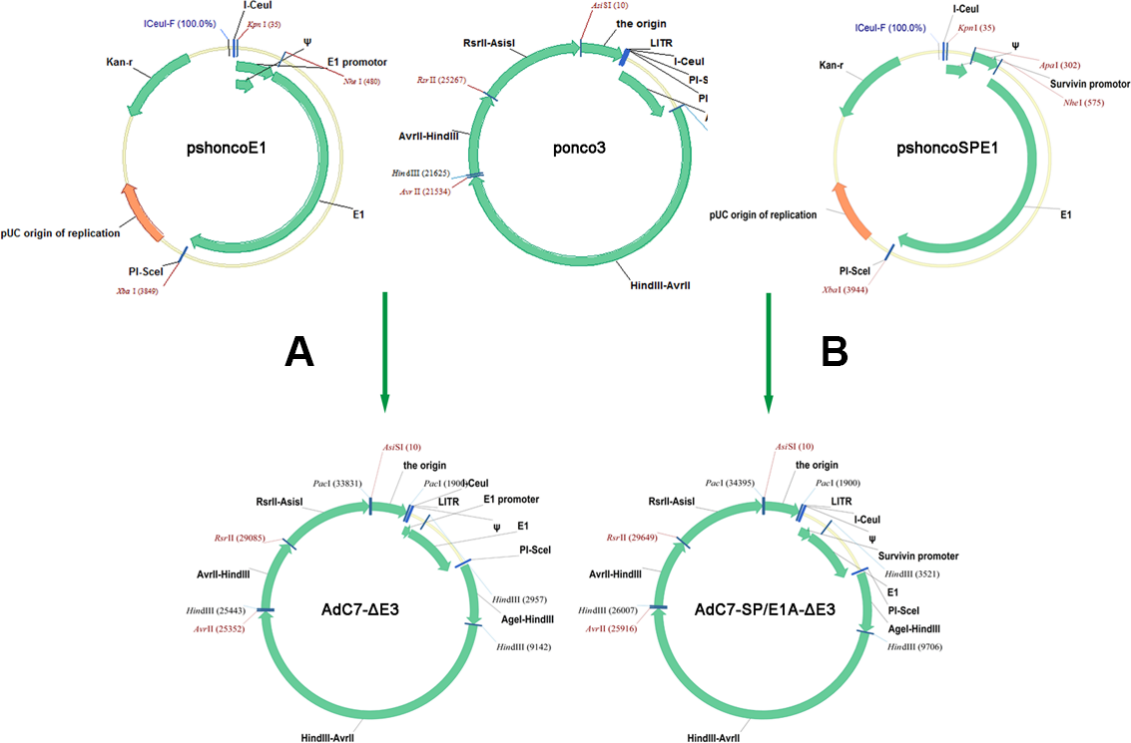
Flowchart of cloning AdC7-ΔE3 and AdC7-SP/E1A-ΔE3 (**A**, **B**) Construction of AdC7-ΔE3 and AdC7-SP/E1A-ΔE3. A fragment containing the E1 region was cut from pshoncoE1 and pshoncoSPE1, and inserted into the ponco3 region between I-Ceu / and PI-Sce /. See Methods for more detail.

The traditional method for generating oncolytic adenoviruses is based on homologous recombination using shuttle vector in bacterial, yeast, and mammalian cells [32]. These systems require a laborious multistep procedure. The present study demonstrates a new oncolytic adenovirus-generating system based on a direct cloning method, which was initially used to produce replication-deficient vector for vaccines [33]. As shown in Figure 9, the system consisted of pshoncoE1 and ponco3 vectors. The vector pshoncoE1 was directly inserted into ponco3 to form oncolytic adenoviruses after manipulation of some viral genome in pshoncoE1. Based on our system, various oncolytic adenoviruses were easily constructed within a short period of time, and the potential of contaminating with unidentified pathogens from chimpanzee could be avoided.

Although the data in Figure 2 suggest that AdC7-SP/E1A-ΔE3 could replicate in tumor cells A549 and SiHa, implying that this virus has the potential to treat such tumors, it could not efficiently kill these tumor cells (Figure 3). In contrast, AdC7-SP/E1A-ΔE3 could not only replicate in but also efficiently kill tumor cells NCI-H508 and Huh7 (Figures 2, 3). Different types of tumor cells vary in proteome, which may determine the differences in the sensitivities of different tumor cells to the same therapy [34, 35]. This could explain the phenomenon that some types of tumor cells such as A549 and SiHa are insensitive to AdC7-SP/E1A-ΔE3 while other types of tumor cells such as Huh7 and NCI-H508 are sensitive. The antagonism to apoptosis induced by AdC7-SP/E1A-ΔE3 in A549 and SiHa could promote the replication of AdC7-SP/E1A-ΔE3, for cells can limit viral replication through death of the infected cell, which is an important cellular defense mechanism against viral invasion [36]. However, NCI-H508 and HuH7 cells infected with AdC7-SP/E1A-ΔE3 could be labeled with Annexin V (Figure 4A), had increasing levels of apoptosis markers (cleaved caspase 3 and PARP) (Figure 4B), and did not experience declining levels of p62 and increasing levels of LC3 II (Figure 4B), suggesting that AdC7-SP/E1A-ΔE3 could kill NCI-H508 and Huh7 cell through apoptosis rather than autophagic cell death, for autophagic cell death is accompanied by the declining of p62 and the increases of LC3 II [37]. Our observations reflect that AdC7-SP/E1A-ΔE3 is distinct in anti-tumor mechanism from AdHu5, which induced autophagic cell death in tumor cells [23]. From the findings on AdHu5 [24] that E1-55k and E4-25k proteins interacted with other cellular proteins to form a ubiquitin ligase complex that degraded the p53 protein, it was speculated that AdC7-SP/E1A-ΔE3 might enable levels of p53 to decline in the infected cells; this hypothesis was validated by data in Figure 5. Moreover, phosphorylation of p53 at serine 46, which is thought to regulate the transcription of apoptosis-inducing genes [38], is absent in cells infected with AdC7-SP/E1A-ΔE3 (Figure 5). These results suggested that apoptosis in infected cells triggered by AdC7-SP/E1A-ΔE3 occurred in a p53-independent manner. The Bcl-2 family proteins, which included antiapoptotic proteins (Bcl-2, Bcl-xl, MCL-1, et al.), proapoptotic proteins (Bim, Bid, PUMA, Bik, Bad, et al.), and the effector proteins Bax and Bak, are well known to interact with each other to regulate apoptosis [39]. In the present study, after infection with AdC7-SP/E1A-ΔE3, NCI-H508 cells had declining levels of antiapoptotic protein MCL-1 and Bcl-2, whereas Huh7 cells had decreased expression of MCL-1and Bcl-xl (Figure 5). E1B 19k protein, the adenovirus Bcl-2 homolog, is required to inhibit human cell apoptosis induced by the infection of adenoviruses [40]. Bik, a potent pro-apoptotic protein, antagonizes anti-apoptotic E1B 19k protein [41]. Our results show that NCI-H508 and Huh7 Cells have increased expressions of Bik after infection with AdC7-SP/E1A-ΔE3. Bad, one of the BH3-only proapoptotic proteins, coupled the death signals to the mitochondrial-mediated pathway, and phosphorylation of Bad at serine 112 was thought to inactivate its proapoptotic function by binding to 14-3-3 scaffold proteins to dissociate Bad from antiapoptotic proteins [42]. Our results show that AdC7-SP/E1A-ΔE3 could enable levels of phosphorylation of Bad at serine 112 to dramatically decline in infected cells, indicating that AdC7-SP/E1A-ΔE3 induced the apoptosis of the infected cells via the Bad-mediated mitochondrial pathway.

In conclusion, we investigated the use of chimpanzee adenoviruses origin- AdC7-SP/E1A-ΔE3 as oncolytic virus for cancer treatment to address the disadvantages of oncolytic adenoviruses based on AdHu5. Importantly, compared to AdHu5, chimpanzee adenoviruses exhibit the lower transduction of hepatocytes [43], which could be explained by the observation that the hexon proteins derived from chimpanzee adenoviruses could not bind to blood coagulation factor X (FX) [17]. Moreover, systemic administration of AdC7-SP/E1A-ΔE3 could also inhibit tumor growth (Figure 8). Oncolytic adenoviruses based on AdC7 are therefore promising candidates for treatment of a variety of tumors, including those of the liver and colon.

## MATERIALS AND METHODS

Cell lines and cell culture. Human embryonic kidney cell line 293, human colorectal cancer cell line NCI-H508, human hepatoma cell line Huh7, human lung cancer cell line A549, cervical carcinoma cell line SiHa, and human embryo lung fibroblastic MRC-5 cells were purchased from the American Type Culture Collection (Manassas, VA). All cell lines were cultured at 37°C in Dulbecco's modified Eagle's medium (Gibco, Grand Island, NY), supplemented with 10% fetal bovine serum (Gibco) and antibiotics.

Adenoviral vectors. A fragment spanning from 809bp to 2691bp of pNEB193 (New England Biolabs, cat. no. N3051S), a fragment spanning from 1 bp to 132 bp of AdC7 (Simian Ad24/Pan7; ATCC, cat no. VR-593) genome, and a fragment spanning from 3027 bp to 4026 bp of AdC7 genome were amplified using following primers, respectively: (sense: actagtcagcctaggacatgcgatcgcaatcaggg gataacgcaggaaagaacatgt, antisense: ttattgatgatgttaattaac tatttttataggttaatgtcatgataataatggtttc); (sense: cctataaaaata gttaattaacatcatcaataatatacctcaaacttttggtgc, antisense: ctcc gcacccgacatagatgcgagctcgctaccttaggaccgttatagttattaaaaacg tcacccgccccgcccctaac); and (sense: agcgagctcgcatctatgtcgg gtgcggagaaagaggtaatgaaatggaaaatgaatcaataaataaacggagac ggttg, antisense: gagaccggtggtccagggcctaccgcgcgcgaaa). These fragments were then fused into new fragments through overlap PCR as previously described [44]. The fused fragment was digested with HindIII and AvrII, and was ligated with a fragment spanning nt 7152–23367 of AdC7 genome with a previously described method [33], forming plasmid ponco1. A fragment spanning nt 31800–36535 of AdC7 genome was amplified (sense: gtggacggctacgattgacggtccgtcacccccttatccagtgaaataaatatc; antisense: cagtgcgatcgcttaattaacatcatcaataatatacctcaaact tttggtgc), digested with AvrII and RsrII, and inserted into plasmid ponco1, forming ponco2. A fragment spanning nt 21533–25346 of AdC7 genome was amplified (sense: gctcaccacgactatttcttctccttg; antisense: tt atttcactggataagggggtgacggaccgtcaatcgtagccgtccaccgactcg c), digested with RsrII and AsisII, and inserted into plasmid ponco2, forming ponco3.

A fragment spanning nt 133–575 of AdC7 genome and a fragment spanning from nt 576–3932 of AdC7 genome were amplified using the following primers respectively: (sense: cgtaactat aacggtcctaaggtagcgaaggtacctacgtggccgtgaggcggagccggttt gca; antisense: ctttcagctagcctttcaaagtgtagatctgactcgc ggcgcg) and (sense: gaaaggctagctgaaagatgaggcacctgaga; antisense cataatgccatttcattacctctttctccgcacccgacatagattcta gattatttggttttcaccgtggcaaccgcggcc), digested with I-CeuI and NheI and with NheI and PI-SceI, respectively, and cloned into digested plasmid pshuttle (Clontech, cat. no. K1650-1) with I-CeuI and PI-SceI, forming pshoncoE1. The survivin promoter was synthesized with recursive PCR [45], and was fused with a fragment from nt 30–290 of pshoncoE1 into the new fragment. The fused fragment was digested with KpnI and NheI, and was cloned into digested pshoncoE1 with KpnI and NheI, forming pshoncoSPE1.

To create the E3-deleted adenovirus AdC7-ΔE3, the fragment containing the E1 region (which was produced by digesting pshoncoE1 with I-CeuI and PI-SceI) was inserted into ponco3, forming the recombinant plasmid pAdC7-ΔE3. Similarly, to create the E3-deleted adenovirus AdC7-SP/E1A-ΔE3, the fragment containing the E1 region (which was produced by digesting pshoncoSPE1 with I-CeuI and PI-SceI) was inserted into ponco3, forming the recombinant plasmid pAdC7-SP/E1A-ΔE3. The recombinant adenoviruses AdC7-ΔE3 and AdC7-SP/E1A-ΔE3 were rescued, propagated in HEK 293 cells, and purified as previously described [33]. AdC7-ΔE1A-ΔE3 of which E1 and E3 region was deleted was constructed as before [16].

E1A mRNA expression assay. Different cell types (∼1 × 10^6^ cells) were infected with 10 MOI of the adenoviruses AdC7-ΔE1A-ΔE3, AdC7-ΔE3, and AdC7-SP/E1A-ΔE3 in Dulbecco's modified Eagle's medium (Gibco, Grand Island, NY) supplemented with 5% fetal bovine serum (Gibco) and antibiotics. Twenty-four hours later, cells were collected, and total cellular RNA was extracted using Trizol Reagent (Invitrogen). RNA (1 μg) from each sample was reverse transcribed to synthesize cDNA using a high-capacity RNA-to-cDNA synthesis kit (Applied Biosystems, Foster City, CA), according to the manufacturer's protocol. E1A mRNA copy number was determined in triplicate experiments by quantitative real-time PCR using the 7900HT Real Time PCR system (Applied Biosystems) as previously described [46]. The E1A primers were sense, gctgctatgaggaatgcttg and antisense, ctttcactccctggttcgat; and the GAPDH primers were sense, ggtttacatgttccaatatgattcca and antisense, atgggatttccattgatgacaag. Data were analyzed using the 7900HT System SDS software (Applied Biosystems).

Adenoviral genome copy number assay. For analysis of adenoviral genome copy numbers, different cell types (∼1 × 10^6^ cells) were infected with 10 MOI of the adenoviruses AdC7-ΔE1A-ΔE3, AdC7-ΔE3, and AdC7-SP/E1A-ΔE3 in Dulbecco's modified Eagle's medium (Gibco, Grand Island, NY) supplemented with 5% fetal bovine serum (Gibco) and antibiotics. Twenty-four hours and 48 h later, cells were collected and, after cells were washed twice with PBS, DNA was purified using the QIAamp Blood Mini kit (Qiagen), following the manufacturer's instructions. Viral genome copy numbers were determined by quantitative real-time PCR, as previously described [47], using the 7900HT Real Time PCR system (Applied Biosystems). The fiber primers were sense, ggtaatggcatagctgcaaa and antisense, aaggtaaggccagtacccaa; and the GAPDH primers were sense, ggtttacatgttccaatatgattcca and antisense, atgggatttccattgatgacaag. Data were analyzed using the 7900HT System SDS software (Applied Biosystems).

Infectious Progeny Production. To determine virus progeny production, 2 × 10^5^ cells were seeded in 6-well plates. Cells were infected with 10 MOI of the adenoviruses AdC7-ΔE1A-ΔE3, AdC7-ΔE3, and AdC7-SP/E1A-ΔE3. After 24 h and 48 h, cells were collected and washed twice with PBS. After three freeze/thaw cycles, the production was determined by TCID_50_ assay on 293 cells.

Cytopathic effect assay. Four tumor cell lines (NCI-H508, Huh7, A549, and SiHa) and a normal cell line (MRC-5) were grown to sub-confluence. These cultures were infected (or not) with AdC7-ΔE1A-ΔE3, AdC7-ΔE3, or AdC7-SP/E1A-ΔE3, at various MOIs. Five days after infection, cells were stained with 2% crystal violet in 20% methanol for 15 min, washed with distilled water, and photographed.

To quantify the cytopathic effect, cells (8 × 10^3^/well) were seeded in 96-well plates and infected (or not) with AdC7-ΔE1A-ΔE3, AdC7-ΔE3, or AdC7-SP/E1A-ΔE3, at various MOIs. Cell survival was assayed at 5 d after infection by an MTT assay, as previously described [48].

Western blot analysis. For detection of the expression of survivin protein in different cell lines, ∼1 × 10^6^ cells from each group were lysed with RIPA buffer containing protease inhibitor cocktail (Roche), and the phosphatase inhibitor cocktail (Roche). For apoptosis assay, NCI-H508 and Huh7 cells (∼1 × 10^6^ cells) were infected with AdC7-ΔE1A-ΔE3, AdC7-ΔE3, or AdC7-SP/E1A-ΔE3, at various MOIs, collected at different time points, and lysed with the above buffers. Equal amounts of total extracted proteins were added to each lane on SDS-PAGE gels (Thermo Scientific, NP0336BOX). Protein expression levels were detected following transfer to PVDF membranes (Millipore, Bedford, MA); incubation with primary antibodies for Survivin, Cleaved Caspase-3, Cleaved PARP, p62, LC3A/B, p53, Phospho-p53 (Ser46), Mcl-1, Bcl-2, Bcl-xl, Bik or Phospho-Bad (Ser112) (Cell Signaling Technology, Danvers, MA); and subsequent incubation with secondary horseradish peroxidase-conjugated antibodies (Sigma-Aldrich, St Louis, MO). The expression of β-actin (Sigma-Aldrich) was measured as a normalization control for protein loading. The signal was detected using a chemiluminescence detection system (GE Health Life Sciences, Pittsburgh, PA).

Flow cytometry assay. Trypsinized cancer cells were washed twice with ice-cold PBS, and incubated with annexin-V-FITC/PI (Sigma-Aldrich) in binding buffer [49]; flow cytometry (FACS) was performed using the BD Fortessa flow cytometer (BD Biosciences, CA, USA); and FACS data were analyzed using the FlowJo software (TreeStar, Ashland, OR, USA).

Xenograft cancer model. All animal experiments were approved by the Institutional Animal Care and Use Committee of Institut Pasteur of Shanghai. Six to eight-week-old female BALB/c nude mice were purchased from Shanghai Laboratory Animal Center (Shanghai, China), and housed under specific pathogen free conditions. Mice were injected subcutaneously (s.c.) on the right dorsal flank with 10^7^ NCI-H508 cells, or 5 × 10^6^ human Huh7 cells mixed with a matrix gel (1:1, BD Biosciences, San Jose, CA). Tumor volume (V) was calculated using the formula: V (mm^3^) = length × width^2^/2. When the tumors reached a volume of 100–150 mm^3^, animals were randomly divided into PBS, AdC7-ΔE1A-ΔE3, AdC7-ΔE3, and AdC7-SP/E1A-ΔE3 groups. A dose of 5 × 10^8^ PFU adenovirus suspended in 50 μL of PBS or 50 μL PBS alone was administrated intratumorally for three times at an interval of 1 d. Tumor volumes were measured using digital calipers every 3 d for NCI-H508 cells, and every 2 d for Huh7 cells.

To examine the antitumor activity induced by AdC7-SP/E1A-ΔE3 via systemic administration, mice were injected subcutaneously (s.c.) on the right dorsal flank with the mixture of 5 × 106 human Huh7 cells with a matrix gel (1:1, BD Biosciences, San Jose, CA). Tumor volume (V) was calculated as above. When the tumors reached a volume of 100–150 mm3, animals were randomly divided into AdC7-ΔE1A-ΔE3 and AdC7-SP/E1A-ΔE3 groups. A dose of 1× 109 PFU adenovirus suspended in 50 μL of PBS was administrated intravenous for three times at an interval of one day. Tumor volumes were measured using digital calipers every two day.

Immunohistochemistry. Tumors were fixed with 1 mL 4% PFA overnight, dehydrated in ethanol, paraffin embedded, and cut into 5 μm sections. DNA fragmentation was determined by TdT-mediated dUTP nick end labeling (TUNEL) as described by the manufacturer (Roche Applied Science, Mannheim, Germany). Fluorescent images were obtained using an EVOS fluorescent microscope (AMG, Bothell, WA).

Statistical analysis. The Prism 5 Software package (GraphPad, La Jolla, CA) was used to draw the graphs and perform statistical analysis. Differences between groups were analyzed by one-way analysis of variance (ANOVA) with Tukey adjustment. For all tests, *P* < 0.05 was considered significant.

## Abbreviations

AdHu5: human adenovirus serotype 5
ChAd: chimpanzee adenoviruses
FX: blood coagulation factor X
SP: survivin promoter
PSA: Prostate-specific antigen
